# Metastasis: imaging shows the way

**DOI:** 10.1186/1470-7330-14-S1-O19

**Published:** 2014-10-09

**Authors:** Hellmut Augustin, Jonathan P Sleeman

**Affiliations:** 1 DKFZ, Im Neuenheimer Feld 280, 69121 Heidelberg, Germany; 2 Medical Faculty Mannheim, CBTM, Ludolf-Krehl-Str 13-17, 68167 Mannheim, Germany; 3 KIT Karlsruhe, Hermann-von-Helmholtz-Platz 1, 76344 Eggenstein-Leopoldshafen, Germany

## 

Metastasis, the life threatening aspect of cancer, is a systemic disease process. Considerable progress has been made in recent years regarding how tumor cells circulating in the blood and lymphatic systems interact with and extravasate into secondary sites, and what determines whether these disseminated tumors cells survive, remain dormant or go on to form macrometastases. New insights into the routes that tumor cells take once leaving the primary tumor have emerged. Novel concepts regarding early seeding of metastases coupled to parallel progression, self-seeding of primary tumors by circulating tumor cells, and the induction of pre-metastatic niches in distant organs by primary tumors have come to the fore. In our presentations we will review these and other paradigm shifts that have taken place over recent years, placing a particular focus on how the use of intravital and other imaging techniques have played a major role in these developments.

